# Rapid and scalable detection of synthetic mRNA byproducts using polynucleotide phosphorylase and polythymidine oligonucleotides

**DOI:** 10.1080/15476286.2024.2363029

**Published:** 2024-06-05

**Authors:** Francis Combes, Thanh-Huong Bui, Frida J. Pettersson, Sjoerd Hak

**Affiliations:** Department of Biotechnology and Nanomedicine, SINTEF, Trondheim, Norway

**Keywords:** mRNA, byproducts, PNPase, assay, high-throughput

## Abstract

Production and storage of synthetic mRNA can introduce a variety of byproducts which reduce the overall integrity and functionality of mRNA vaccines and therapeutics. mRNA integrity is therefore designated as a critical quality attribute which must be evaluated with state-of-the-art analytical methods before clinical use. The current study first demonstrates the effect of heat degradation on transcript translatability and then describes a novel enzymatic approach to assess the integrity of conventional mRNA and long self-amplifying mRNA. By first hybridizing oligo-T to the poly(A) tail of intact mRNA and subsequently digesting the unhybridized RNA fragments with a 3’-5’ exoribonuclease, individual nucleotides can be selectively released from RNA fragments. The adenosine-based fraction of these nucleotides can then be converted into ATP and detected by luminescence as a sensitive indicator of mRNA byproducts. We developed a polynucleotide phosphorylase (PNPase)-based assay that offers fast and sensitive evaluation of mRNA integrity, regardless of its length, thus presenting a novel and fully scalable alternative to chromatographic-, electrophoresis-, or sequencing-based techniques.

## Introduction

The instructive information necessary for producing proteins is fully contained within mRNA molecules. This premise led to the recent development of mRNA vaccines and therapeutics as a platform technology to produce a new class of medicines comprising a cap, untranslated regions, a coding sequence, and a poly(A) tail. However, mRNA is known for being unstable when exposed to water, particularly at high pH, high Mg^2+^ concentration, and heat [1]. This instability is caused by RNA hydrolysis, which occurs when the ribose 2’hydroxyl loses a proton and the now negatively charged 2' oxygen attacks the adjacent 3' phosphodiester bond [1]. The resulting cleavage event can subsequently render the mRNA untranslatable for ribosomes. Hence, assessing mRNA integrity is an important quality control for downstream medical applications. High performance liquid chromatography (HPLC) and capillary gel electrophoresis (CGE) are currently considered industry standards for assessing mRNA integrity. Nevertheless, identifying minor changes in mRNA integrity presents an ongoing challenge due to e.g. peak broadening and unpredictable intramolecular structure formation, particularly for long mRNA molecules. To standardize electropherograms and enable higher resolution fragment separation, CGE protocols for RNA analytes often employ strongly denaturing conditions including shortly heating the RNA at temperatures ≥ 70°C [2,3]. Heating RNA is, however, associated with accelerated rates of hydrolysis and hence results in induced degradation prior to analysis, potentially leading to false interpretations. The current study aims to provide an alternative to separation-based analysis of mRNA integrity by sensitively detecting the presence of mRNA fragments that lack a poly(A) tail. Such fragments are directly related to abortive, truncated, complementary, readthrough and/or degraded transcripts and may therefore provide an intuitive readout. More specifically, we demonstrate how combining oligonucleotides and PNPase (an ssRNA-specific 3'-5' exoribonuclease) can selectively release nucleoside diphosphates from RNA degradation fragments. Further enzymatic conversion of the adenosine diphosphate (ADP) fraction into luminescence allows rapid, sensitive, and scalable detection of mRNA byproducts in a sample.

## Results

### Heating mRNA induces more strand breaks than indicated by CGE

**Figure 1.**
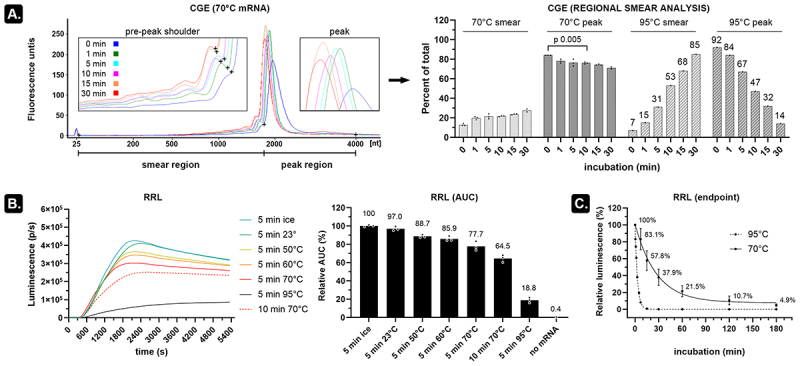
Subtle temperature dependent mRNA degradation decreases the number of functional transcripts.

CGE can be performed on microchips with high sensitivity, but this requires a formamide-based method which involves heating mRNA for 10 min at 70°C [3]. In our hands, however, regional smear analysis indicated that this heat denaturing step results in a slight decrease in relative peak area (84% to 76%) and an associated increase of the pre-peak smear area from 12.7% to 21.7% on an Agilent 2100 Bioanalyzer system as compared to non-denaturing conditions (Figure 1A and S1). Longer heating times resulted in more pronounced peaks with a narrower base (indicating uniformization of RNA structures) but also caused a peak shift and increased the pre-peak shoulder height. It is unclear whether this shoulder represents truncated transcripts, degraded transcripts, or faster migrating alternative structures of the full-length transcript (known as conformers [4]). A clearer degradation pattern can be seen when heating mRNA at 95°C with a relative peak area decreasing from 92% to 14% and a concurrent smear increase from 7% to 85% (Figure 1A and S1). Subjecting the firefly luciferase (fluc)-encoding mRNA (~2 kb) to a kinetic rabbit reticulocyte lysate (RRL) assay confirmed mRNA integrity loss due to heating as a pronounced decrease in protein output (Figure 1B). A subtle decline of functional transcripts already occurred after incubating the mRNA for 5 min at room temperature and even faster decline was seen when heating the mRNA in 90% formamide (Figure S2). Assessment of heated mRNA at multiple incubation periods indicated that over 95% of the functional transcripts disappeared after 3 h incubation at 70°C (t½ = 19.0 min, Figure 1C). In contrast, virtually all functional transcripts were removed after 10–15-min incubation at 95°C (t½ = 2.1 min, Figure 1C) despite that a peak at the position of unheated (‘intact’) mRNA (32%) was still visible on CGE (Figure 1a and S1). These data thus indicate that incubating mRNA at room temperature (or higher temperatures) for even short periods of time decreases the number of protein-encoding transcripts. Chromatographic or electrophoretic analysis of heat-denatured mRNA consequently needs to be evaluated with caution. Alternatively, analysis of non-denatured mRNA yields hard-to-interpret migrating broad peaks with shoulders and smears that do not necessarily relate to the level of functional transcripts in a sample.

A) mRNA encoding firefly luciferase (fluc) of about 2 kb long was incubated at either 70°C or 95°C for 0–30 min and subsequently evaluated using CGE (200 ng per lane). The electropherogram depicts the mRNA samples that were incubated at 70°C with more detailed views on the pre-peak shoulder and peak migration (insets). Regional boundaries were manually placed at the ‘+’ signs to delineate the smear region from the peak region. The smear analysis for these regions is depicted in the bar graph on the right (3 technical replicates). Additional electropherograms can be found in Figure S1. b) Fluc mRNA (500 ng) was incubated in nuclease free (NF)-water at the indicated temperatures for either 5 or 10 min. These samples were then used in a rabbit reticulocyte lysate (RRL) assay which was continuously measured on a luminometer in the presence of substrate D-luciferin (3 technical replicates). Both the continuous measurements and the derived AUC values indicate that a decrease in the number of functional transcripts is already noticeable after incubating mRNA for 5 min at room temperature (23°C) and that incubating mRNA for 10 min at 70°C substantially decreases expression even further. c) For each indicated timepoint, fluc encoding mRNA (500 ng) was heated at either 70°C (3 independent experiments, error bars depict SD) or 95°C (*n* = 1) in PBS and subsequently used in a 1-h RRL assay and a luminescence readout of the produced fluc proteins.

### Combining exoribonuclease and oligo-T30 allows assessment of mRNA fragments

**Figure 2.**
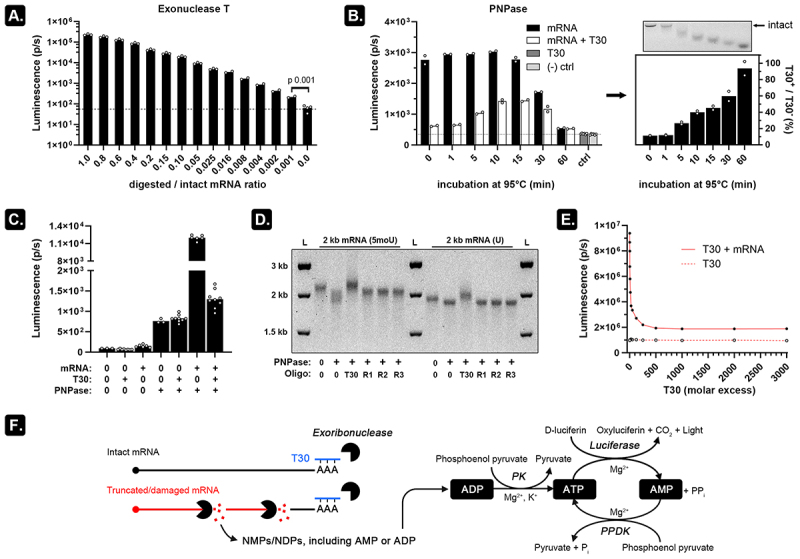
PNPase and oligo-T30 can be combined to selectively release measurable ADP from mRNA fragments.

An RNA molecule is a linear polymer of (ribo)nucleoside monophosphates (NMPs) which on average comprises 25% adenosine monophosphate (AMP). This AMP fraction can be liberated by digesting the RNA analyte with an exoribonuclease, enzymatically converted into ATP, and finally detected using commercial kits. To test this approach, we digested intact mRNA with exonuclease T, an enzyme that catalyses the release of nucleoside monophosphates from ssRNA in the 3’to 5’ direction. By mixing this digested mRNA with intact mRNA in different ratios and subsequently measuring the AMP content using the Kikkoman Lucipac^TM^ A3 reagent mix [5], we noticed that addition of as little as 0.1 weight% digested mRNA to intact mRNA resulted in a signal increase compared to undigested (intact) mRNA. This approach is therefore highly sensitive for detecting small AMP variations in RNA samples (Figure 2A). Since degraded mRNA contains more individual mRNA fragments due to hydrolytic cleavage, we reasoned that RNA degradation should consequently result in more signal generation after adding a 3'-5' exoribonuclease (as more 3' ends will be available for the enzyme), especially if that enzyme does not fully degrade the mRNA fragments.

A) mRNA encoding fluc was digested for 1 h by Exo T and mixed with undigested (intact) mRNA at the indicated fractions. We detected a differential luminescence signal in mRNA mixed with as low as 0.1% digested mRNA (paired t-test p 0.001) upon incubating these samples with the Lucipac A3 reagents (the dots represent technical replicates). b) When using PNPase, the signal from the heat degraded mRNA only starts to drop when mRNA was degraded for more than 15 min. In contrast, in the presence of 500-fold molar excess T30, excessive signal generated at the poly(A) tail is suppressed. The average signal derived from mRNA in the presence of T30 increases relatively to the signal obtained in the absence of T30 (depicted in the right graph as T30+/T30- ratio). When loaded on agarose GE, the heat degraded mRNA fragments also demonstrate a concurrent decrease in size. c) Annealing 500-fold molar excess T30 to intact mRNA quenches PNPase-mediated luminescence. The background luminescence is mainly associated with the PNPase homotrimer itself (*n* ≥ 6). d) One-hour PNPase digestion shortens both modified (5moU) and unmodified (U) mRNA. Addition of T30 oligos but not random oligos (R1, R2 and R3) prevents this shortening, as indicated by agarose GE (DNA ladder is indicated with ‘L’). e) Addition of T30 to the PNPase digestion reaction exhibits a concentration-dependent suppression of luminescence from intact mRNA with saturation reached at 1000-fold molar excess. f) Representation of the enzymatic RNA degradation assay: an exoribonuclease is blocked by T30 oligos at the poly(A) tail of intact mRNA (blue) but can digest truncated or damaged mRNA fragments (red). This liberates AMP or ADP which can subsequently be converted to ATP and measurable light.

**Figure 3.**
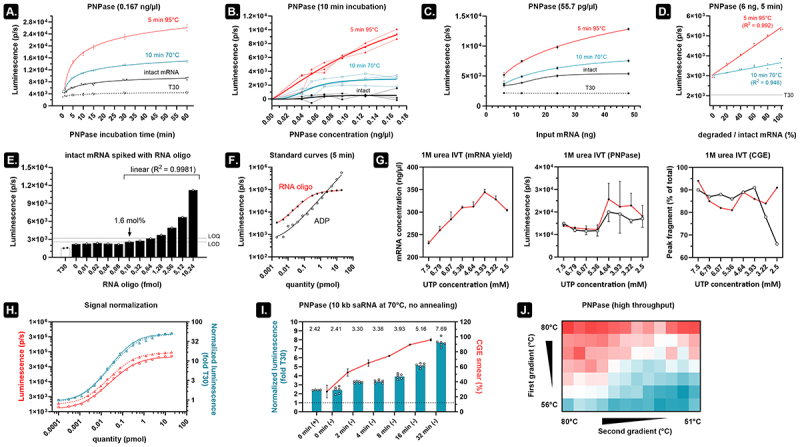
An optimized PNPase assay is fast, sensitive, and does not depend on the size of the mRNA analyte.

To test this hypothesis, we used polynucleotide phosphorylase (PNPase), a phosphorolytic 3'-5' exoribonuclease that is compatible with 2',3'-cyclic phosphate, 2'-phosphate, or 3'-phosphate residues which are typically present after hydrolytic cleavage [6]. PNPase digests RNA into diphosphate nucleotides (NDPs) of which the ADP fraction is efficiently converted into luminescence (Figure S3). To prevent signal generation from ssRNA-specific PNPase digestion of intact mRNA, we tried annealing a 500-fold molar excess of T30 (dT30) deoxyribonucleotides to the poly(A) tail prior to PNPase digestion (Figure 2B). DNA:RNA hybridization almost completely suppressed the luminescence to background levels on intact mRNA. As 95°C degradation time was prolonged, an increasing number of hydrolytic cleavage events exposed more unhybridized 3' ends which can be digested by PNPase. This increases the luminescence signal until 10–15 min of heat degradation (which corresponded to extreme degradation as seen on Figure 1C). The calculated relative luminescence (signal ratio of mRNA T30+/mRNA T30-) indicates that the relative number of digestible mRNA fragments increases over time. The concurrent shortening of the mRNA fragments can also be seen on the accompanying agarose GE image (right panel on Figure 2B). The decreased luminescence at 30–60-min incubation at 95°C can likely be attributed to the accumulation of very short (<7-9nt) RNA fragments which cannot be digested by PNPase [7]. Figure 2C identifies PNPase as the main source of the observed background luminescence, likely coming from residually bound ADP to the PNPase homotrimer. We tried purifying the His-tagged PNPase using 4°C (NH_4_)_2_SO_4_ precipitation overnight, a 3 kDa MW cut-off modified PES column, and cobalt-based purification, but were unable to adequately reduce the background signal (data not shown). Agarose GE shows that inhibition of mRNA digestion by PNPase only occurs when T30 oligos are added to the reaction and not when randomized 30 nt long oligonucleotides (R1-R3) are used (Figure 3D) thereby demonstrating that the impaired recognition of mRNA by PNPase relies on specific interactions between the poly(A) tail and the T30. Further T30 dilution experiments show that at about 500–1000-fold molar excess, near to maximal suppression of PNPase activity is observed (Figure 2E). Hybridizing a 1000-fold molar excess of T30 to the poly(A) tail followed by PNPase digestion thus allows selective release of NDPs of which the ADP fraction can be measured by luminescence (Figure 2F).

A) 12 ng heat degraded mRNA (2 kb) was subjected to the PNPase assay (1000-fold T30, 0.167 ng/µl PNPase) over the indicated time period. Most of the signal is generated within 15 min of incubation. b) 6 ng of heat degraded 2 kb mRNA was evaluated in the PNPase assay (10 min digestion) using varying concentrations of PNPase (x-axis) in 3 independent experiments performed on different days. The increasing background luminescence associated with the PNPase was subtracted from all depicted values. c) Using a final concentration of 55.7 pg/µl PNPase, higher input mRNA increases the signal difference between intact and heat degraded mRNA. Three technical replicates and error bars represent SD. d) Even at 6 ng input mRNA and only 5 min of incubation, a linear increase in luminescence is seen when gradually more degraded mRNA is present. e) 10 fmoles 2 kb mRNA was spiked with increasing quantities of a 60-mer RNA oligo and evaluated in the PNPase assay (10 pmol T30, 55.7 pg/µl PNPase, 10 min digestion). The LOD (3.3*SD) and LOQ (10*SD) are estimated by aggregating the measurements below 0.16 fmol RNA oligo. Luminescence values from 0.16 fmol to 10.24 fmole demonstrate excellent linearity. f) A standard curve of the 60-mer RNA oligo and ADP was made in absence of mRNA (but in the presence of T30). In the linear range of both graphs (10-100 fmole), 4-5 ADPs per RNA oligo are released. g) Twenty-four 1 M urea supplemented IVT reactions were performed using 8 different UTP concentrations (3 replicates). After incubating with DNase, the resulting mRNA was purified using silica beads and then subjected to UV spectrophotometry and PNPase assays (two independent repeats performed on different days, error bars depict SEM). The CGE analysis was also performed on two different days, but the three replicates were combined to form each sample. h) Dividing the luminescence signal of two independently repeated standard curves of a 60-mer RNA oligo (left y-axis) by their respective T30-only controls (right y-axis) minimizes variability. i) Cellulose-purified 10 kb saRNA was heat degraded at 70°C for the indicated time periods and analysed on non-denaturing CGE (right Y-axis, two technical repeats, 200 ng per lane, error bars depict SD). When omitting the annealing time and evaluating the heat degraded saRNA in the PNPase assay (5 min digestion, performed in six independent replicates on multiple days), we can detect saRNA fragments with similar sensitivity as CGE and minimal variability (left Y-axis; values are noted above each bar; (+) indicates 10 min annealing at room temperature, whereas (-) indicates where the annealing step was omitted). j) Ten femtomoles of Cre recombinase mRNA (~1 kb, Trilink) was distributed in a 96 well plate and first subjected to a 5-min heat gradient (80-56°C) along the vertical direction in a thermocycler. We then applied a 5-min heat gradient (80-51°C) along the horizontal direction. The heat map represents T30-normalized values from a PNPase assay (1000-fold molar excess T30, no annealing step, 5 min PNPase digestion).

### An optimized PNPase assay rapidly and sensitively reports on mRNA quality, irrespective of mRNA size

Incubating intact and degraded mRNA at different times and subsequent luminescence measurements demonstrate that samples containing only T30 do not exhibit increased signal over time. These samples are therefore a good indicator of background luminescence (Figure 3A). In addition, the rapid release of ADP results in clear signal differences already after a few minutes of incubation (Figure 3A) and with a minimal PNPase concentration (Figure 3B). The low variation seen in the latter experiment suggests that the PNPase assay is very reproducible. Using a fixed enzyme concentration (55.7 pg/µl) but varying the input mRNA from 6 ng (~10 fmol) to 48 ng shows that more mRNA leads to higher sensitivity (Figure 3C). Nevertheless, even at 6 ng input mRNA and 5 min of PNPase digestion, it is possible to discriminate different fractions of intact mRNA mixed with degraded mRNA (Figure 3D). Next, we set out to see how the PNPase assay performs in detecting well-defined quantities of mRNA fragments. To this end, we selected a 60 nt-long sequence (50% GC) from the luciferase gene and ordered it as unmodified RNA oligos. Ten femtomole intact fluc mRNA (2 kb) was spiked with increasing amounts of the 60-mer RNA oligo and annealed to a 1000-fold molar excess T30 followed by 5 min of PNPase digestion (55.7 pg/µl) and luminescence measurement (Figure 3E). We can determine the standard deviation (SD) of the background luminescence by aggregating the signals that lack visually detectable differences (0–0.08 fmol RNA oligo). This allows us to estimate an LOD (3.3*SD) between 1 and 1.6 mol% and an LOQ (10*SD) between 6.4 and 12.8 mol% [8]. The data indicates that addition of 1.6 mol% to the 10 fmoles mRNA could already be discriminated above the LOD with excellent linearity (R^2^ = 0.9981). In addition, using standard curves of both the 60-mer RNA oligo and ADP in the presence of 10 pmol T30 indicated that, within the linear range, on average 4.5 ADP molecules are released per RNA oligo (Figure 3F). This finding makes it in theory possible to employ ADP dilutions to estimate the number of free 3' ends in a sample.

We subsequently wanted to test how the PNPase assay performs in finding the optimal IVT reaction conditions. In our recent publication [9], we demonstrated that supplementing 1 M urea to the IVT reaction decreases the level of mRNA byproducts. However, the UTP concentration is an important determinant of the IVT reaction [10] and addition of urea likely influences its reaction optimum. We performed 24 IVT reactions (8 different UTP concentrations and 3 technical replicates) which were then evaluated for mRNA yield and mRNA quality (Figure 3G). Our findings indicate that higher UTP concentrations result in higher quality mRNA at the cost of lower yield. Two independent PNPase assays (performed on different days) show that using 5.36 mM UTP during urea IVT provides a good trade-off between high yield and high quality. In agreement with these results, CGE indicated a peak fragment of about 80–90% with highest average integrity seen at high UTP concentrations and declining integrity seen at low UTP concentration.

Most variation between independent repeats of the PNPase assay on a 60-mer RNA oligo can be removed by normalizing the luminescence values by the corresponding T30-only signal (Figure 3H). We also omitted the annealing step and detected similar sensitivity for detecting 10 kb self-amplifying (sa)RNA degradation using CGE and the PNPase assay (Figure 3I and S4). Lastly, using commercially available Cre recombinase mRNA (Trilink), we demonstrated that the PNPase assay is amenable to higher throughput setups such as demonstrated in Figure 3J.

## Discussion

The presented PNPase-based assay describes a scalable and cost-efficient alternative to separation-based techniques by directly linking the level of mRNA byproducts in a sample to luminescence readout. We demonstrate that reproducible signal can be generated from both short (1–2 kb) mRNA and long (10 kb) saRNA after only 5 min of incubation with detection sensitivities that currently match those of a Bioanalyzer 2100 CGE system.

It is compelling to see the marked effect of mild temperature increase on RNA integrity (Figure 1), even at incubation times as short as 5 min. Many protocols in the RNA research field – including routine slab-gel GE – start with a short denaturing step to remove the RNA secondary structure. In addition, mRNA purification [11] and even analytical methods to quantify changes in mRNA integrity (e.g. RT-PCR [12], CGE [3], or HPLC [11,13] require a certain amount of time to run the assay. This is often conducted at elevated temperatures and hence induces ‘intra-assay’ RNA cleavage events. At the same time, heating affects the distribution of alternative mRNA structures (known as conformers) which exhibit an altered migration pattern on CGE [4]. Correctly identifying the nature of the various peaks on CGE or HPLC in both denaturing and non-denaturing conditions is therefore challenging as they can either be abortive transcripts, truncated transcripts, complementary dsRNA, readthrough transcripts, mRNA degradation fragments, or conformers of intact mRNA. Moreover, separation-based techniques lose resolution as mRNA length increases, exacerbating this challenge in e.g. very long saRNA [14]. Although more advanced CGE systems [15] or long-read Nanopore sequencing [16] show great promise in more sensitively identifying changes in mRNA integrity, these methods require a considerable amount of time, expertise, and financial resources to run. Moreover, both methods employ mRNA heating which, as seen in Figure 1, results in underestimating the initial mRNA integrity. In the current report, we demonstrated that selective enzymatic digestion of RNA fragments (lacking a poly(A) tail), using minimal resources and no excessive heating, results in an unambiguous readout: any signal higher than the background luminescence is due to the presence of untailed mRNA fragments, which are all unwanted byproducts. However, byproducts such as 3' loop-back dsRNA and transcripts with shorter poly(A) tails are unlikely to be detected by the PNPase assay [9,17]. It is also important to note that the PNPase assay requires incubation at 37°C and hence also introduces some intra-assay cleavage events. These events can be estimated as a steady increase (1.30%) in luminescence between e.g. 30–60 min digestion on Figure 3A. Based on this result, the effect of intra-assay cleavage events in a 5-min digestion step should be minimal. Moreover, the findings in Figure 3A suggest the possibility of reducing the assay duration even further.

PNPase is the main source of background luminescence (Figure 2D) [18]. We therefore settled on using a low concentration of PNPase (55.7 pg/µl) in combination with 1000-fold molar excess T30 oligonucleotides which do not require a separate annealing step (Figure 3i). We used only 5 min of incubation to detect mRNA degradation fragments with similar sensitivity as the Bioanalyzer 2100 system (Figure 3I). Furthermore, signal normalization reduced inter-experimental variability (Figure 3H). If needed, higher sensitivity for detecting mRNA byproducts can be achieved by e.g. prolonging PNPase digestion time (Figures 3A–C). The PNPase assay can thus be optimized to the needs of individual users to, for example, define mRNA quality threshold values. These thresholds can then be referenced throughout a synthetic mRNA’s period of use (e.g. a value below 1.5 × T30 defines an mRNA of excellent quality, whereas a value above 2.0 × T30 might be used to indicate unacceptable quality for downstream applications). Such relative scoring of mRNA integrity is helpful in higher throughput setups. Figure 3G, for example, shows that the PNPase assay can be used to find the optimal UTP concentration for a certain template. In this experiment, we weighted the IVT concentration of each NTP around 5 mM in accordance to its relative nucleoside presence in the template (30.6% A, 29.6% C, 25.5% G) while varying the UTP concentration from 2.5–7.5 mM. In the weighted scheme, UTP would require a concentration of 2.87 mM (14.3% U), but the PNPase assay suggests that increasing this concentration to at least 5.36 mM results in a lower level of mRNA byproducts (Figure 3G). Although higher UTP concentrations resulted in similar mRNA quality, excess UTP should be avoided as this is associated with higher levels of dsRNA [10]. Exhaustive optimization of the IVT reaction should evaluate all NTPs, Mg^2+^, pyrophosphatase, RNA polymerase, pH, temperature, etcetera in a comprehensive design of experiments approach. This was beyond the scope of the current investigation, but we do show that the PNPase assay is amenable to high throughput setups (Figure 3J) and that the assay can be employed to assess very long mRNA constructs such as 10 kb self-amplifying mRNA (Figure 3I). Although saRNA vaccines were recently approved for clinical use in Japan [19], its large size and complex structure makes saRNA notoriously challenging to analyse using conventional bioanalytical methods [14]. Compared to shorter mRNA, our experiments also exposed a higher susceptibility of 10 kb saRNA for strand breaks. This finding is in accordance with earlier reports that estimated a ~ 3-fold shorter half-life of 12 kb saRNA compared to 4 kb mRNA [20].

Autocatalytic hydrolytic cleavage of RNA strands produces 2',3'-cP residues [21] and such residues can also be formed after enzymatic cleavage [22]. To avoid elaborate assay modifications, we have focused on using the 2',3'-cP-compatible PNPase. Indeed, in the presence of 10–20 mM inorganic phosphate, cyanobacterial PNPase removes ribonucleotides in a 3'-5' direction but can only bind fragments of at least 7–9 nt long [7]. This size requirement likely caused the signal decrease when mRNA was highly degraded at 95°C (Figure 2B). It is also important to note that the PNPase assay only informs on bulk mRNA quality and does therefore not replace chromatographic methods. Another limitation of the assay in its current form is that it can only be used on synthetic mRNA containing a poly(A) tail and is limited to relative quantification. Despite these shortcomings, however, we envision that the speed, low cost, and scalability of the PNPase assay offers a valuable alternative to current resource-intensive techniques.

## Materials and methods

### mRNA and RNA oligos

Capped 5moU-modified mRNA encoding firefly luciferase (fluc) was either purchased at Trilink Biotechnologies (L-7202) or produced via NEB’s HiScribe® T7 high yield RNA synthesis kit (E2040) using non-modified uridine where indicated. Stock mRNA was typically diluted to 20 ng/µl in nuclease-free water before initiating heat degradation in a thermocycler or heating platform for the indicated time and temperature. After heating, the RNA was immediately transferred to ice and shortly centrifuged before further use. Routine quality control of produced mRNA and agarose gel-based experimental procedures used in this study employed 0.8–1% agarose gels in TAE supplemented with 1% bleach to inactivate RNases [23]. GelRed® was used to stain the RNA (Merck SCT122), and NEB’s 1 kb plus DNA ladder (N3200) was used as control. For the IVT reactions in Figure 3g, we used 50 ng linearized template DNA as input and performed urea supplemented IVT reactions as previously described [9] but varied the UTP concentration from 2.5–7.5 mM. After DNase I digestion (10 min at 37°C), we purified the mRNA using the RNAClean XP (Beckman Coulter) silica-coated magnetic beads according to the manufacturer’s instructions. Next, a Tecan Spark plate reader was used to determine the concentration of the mRNA (UV spectrophotometry at 260 nm), and each sample was brought to the same concentration by adding nuclease-free dH_2_O. HPSF-purified T30 and 30 nt-long random oligos R1–3 (respectively TTTACCGCAACTACACCTAACTGAGATACT, TTAGATAACCGGATACAGTGACTTTGATAG, CTGCGTATGGAGGAAGGAACTTTTGCGTGT) were ordered from Eurofins. Self-amplifying mRNA (9654 nt, including a poly(A) tail of 40 nt) was kindly provided by Pieter Vervaeke (Niek Sander’s lab at Ghent University) after in vitro transcription (NEB E2040) and co-transcriptionally capping with cleancap AU (Trilink N7114). Cellulose purification of the saRNA was performed according to Baiersdörfer et al. [24]. The 5moU-modified Cre recombinase mRNA used in Figure 3J was purchased at Trilink Biotechnologies (#L-7211). The 60-mer RNA oligo (UCCAGAAGAUCAUCAUCAUGGACAGCAAGACCGACUACCAGGGCUUCCAGAGCAUGUACA) is identical to a 50% GC region in the used fluc mRNA and was ordered from IDT.

### Capillary gel electrophoresis

Fluc encoding mRNA (~2 kb) or saRNA (~10 kb) was either left intact or heat degraded as described above. Unless specified otherwise, we loaded 200 ng mRNA per lane onto an Agilent Bioanalyzer 2100 system using the Agilent RNA 6000 Nano kit (#5067–1511) according to the manufacturer’s instructions. Non-denaturing conditions excluded the mRNA sample heating step prior to loading on the chip, whereas samples in denaturing conditions were heated for 2 min at 70°C prior to loading. Electropherogram smear analysis was performed on the Agilent 2100 Expert software using manually placed regional borders as indicated in Figure 1.

### Cell-free translation

Five hundred nanogram mRNA was dissolved in 13 µl nuclease-free (NF) water and combined with 10 µl nuclease-treated rabbit reticulocyte lysate (RRL, Promega #L4960) containing all amino acids. Twenty-one microlitre of this mixture was then incubated for 60–90 min at 30°C. One microlitre D-luciferin substrate (0.75 mg/ml) was either added to this reaction (kinetic analysis [25]) or added after the 30°C incubation (endpoint analysis). Luminescence readouts were performed in either white 96-well or white 384-well plates on a Tecan Spark plate reader.

### Exo T reaction

mRNA was heat degraded as indicated and digested with Exo T (NEB # M0265) in NEBuffer 4 (NEB #B7004S) according to the manufacturer’s instructions. This mixture was incubated for 1 h at 37°C on a thermocycler before transferring to ice. Five microlitre of this reaction was transferred to a white 96-well plate (ThermoFisher #136101) already containing 25 µl NF-water and 30 µl 1:3 diluted LuciPac™ A3 reagent mix (Kikkoman #60365). Luminescence readout occurred on a Tecan Spark plate reader.

### PNPase reaction

mRNA was heat degraded as indicated and diluted to 4 ng/µl. 10 fmoles mRNA (unless stated otherwise) was then mixed with 1000-fold molar excess oligo-T30 and NF-water in a total volume of 14 µl per reaction. Annealing was performed for 10 min at room temperature followed by brief centrifugation (this annealing step was eventually omitted). Next, the annealed mRNA:T30 was mixed with 3 µl *Synechocystis sp*. PNPase (Sigma #N9914) at 55.7 pg/µl (unless stated otherwise), 3 µl buffer A (50 mM MgCl_2_, 100 mM KCl, 500 mM Tris-HCl pH 8.5), 7 µl NF-water, and 3 µl 10× PBS as a source of inorganic phosphate (ThermoFisher AM9625). Incubation of the PNPase digestion reaction occurred at 37°C for the indicated period of time (5 min after optimization). After a short centrifugation step, 75 µl NF-water was added to each tube to facilitate the transfer of 50 µl reaction mixture to a white 96-well plate. Finally, 15 µl 1:3 diluted LuciPac™ A3 reagent mix (Kikkoman Biochemifa #60365) was added per well before measuring the luminescence after 3–5 min on a Tecan Spark plate reader.

## Supplementary Material

Combes2024_PNPaseAssay_supplement.docx

Figure Supplement.tif

## Data Availability

The authors confirm that the data supporting the findings of this study are available within the article and its supplementary materials.
