# Supplementary material for: Rapid and scalable detection of synthetic mRNA byproducts using polynucleotide phosphorylase and polythymidine oligonucleotides
Source: RNA Biol. 2024 Jun 5;21(1):1–8. doi: 10.1080/15476286.2024.2363029 (PMC11155706; doi:10.1080/15476286.2024.2363029)
Supplement: Combes2024_PNPaseAssay_supplement.docx [file KRNB_A_2363029_SM1797.docx]

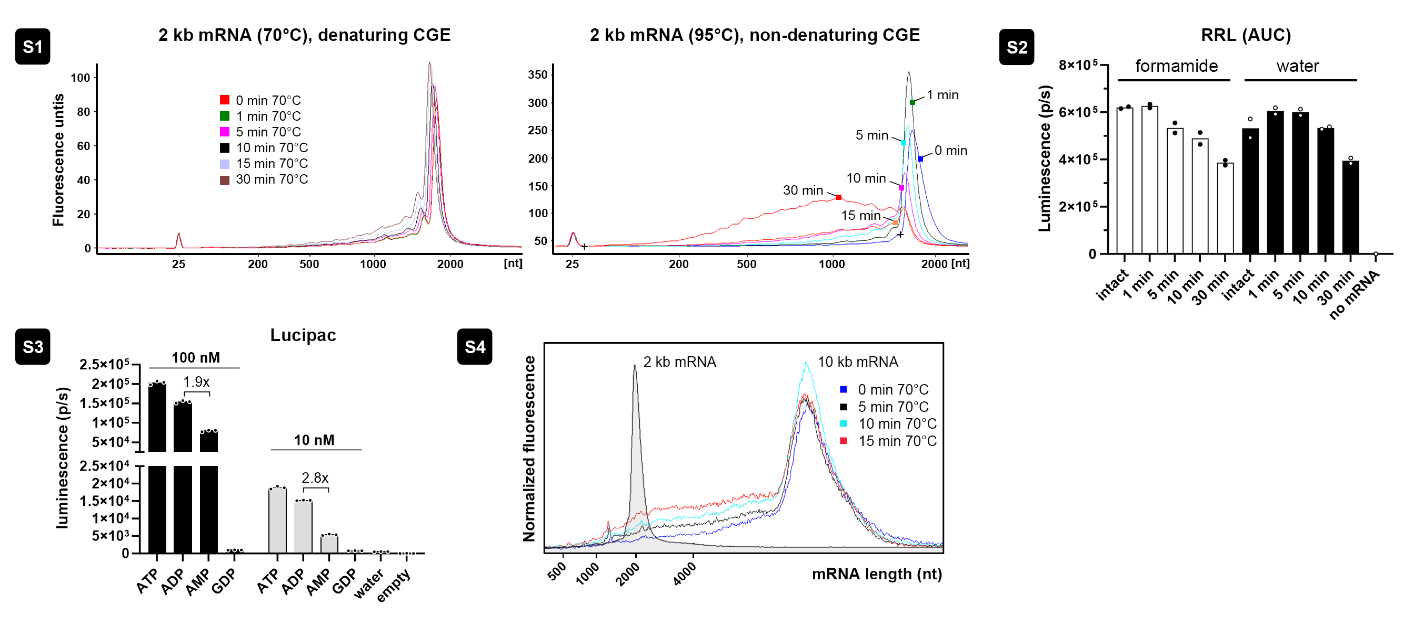


***Supplement Figure. S1)*** *Left graph:* *2 kb mRNA was heated at 70°C for the indicated periods of time. Two hundred nanograms of each sample was loaded on a Bioanalyzer 2100 system in denaturing conditions (heating 2 minutes at 70°C prior to loading, as indicated by the manufacturer). Although the peak width is narrower in denaturing conditions, a pre-peak smear with a shoulder/smaller peak is still present. Right graph: 2 kb mRNA was heated at 95°C for the indicated periods of time. Two hundred nanograms of each sample was loaded on a Bioanalyzer 2100 system in non-denaturing conditions. The “+” signs indicate the regional limits used for smear analysis (the same method was used as in Figure 1A). The electropherogram shows that a peak is still present in the 10-, 15-, and 30-minutes samples that were heated at 95°C even when no luminescence could be detected on RRL (Figure 1C).* ***S2)*** *mRNA encoding fluc was dissolved in deionized water or in 90% formamide. These samples were then heated at 70°C for the indicated periods of time followed by purification on a silica column (NEB T2047L) and levelling the samples to an equal concentration after UV spectrophotometry. Next, 500 ng of each sample was used in a 2-hour µl RRL reaction in the presence of D-luciferin substrate (65 ng/µl final concentration) according to the manufacturer’s instructions (Promega). The bars represent the AUC of the total luminescence signal over 2 hours of incubation. The results suggest very similar degradation rates in 90% formamide versus water.* ***S3)*** *ADP generates 1.9-2.8 times more luminescence than AMP in the Lucipac A3 assay. Exoribonucleases releasing NDPs are therefore expected to be more sensitive than NMP-releasing exoribonucleases.* ***S4)*** *Cellulose-purified 10 kb saRNA was heat degraded at 70°C for the indicated time periods and analysed on non-denaturing CGE using 200 ng per lane. An intact mRNA of 2 kb was added for reference.*
