# Supplementary figures and images for: Rapid and scalable detection of synthetic mRNA byproducts using polynucleotide phosphorylase and polythymidine oligonucleotides
Source: RNA Biol. 2024 Jun 5;21(1):1–8. doi: 10.1080/15476286.2024.2363029 (PMC11155706; doi:10.1080/15476286.2024.2363029)

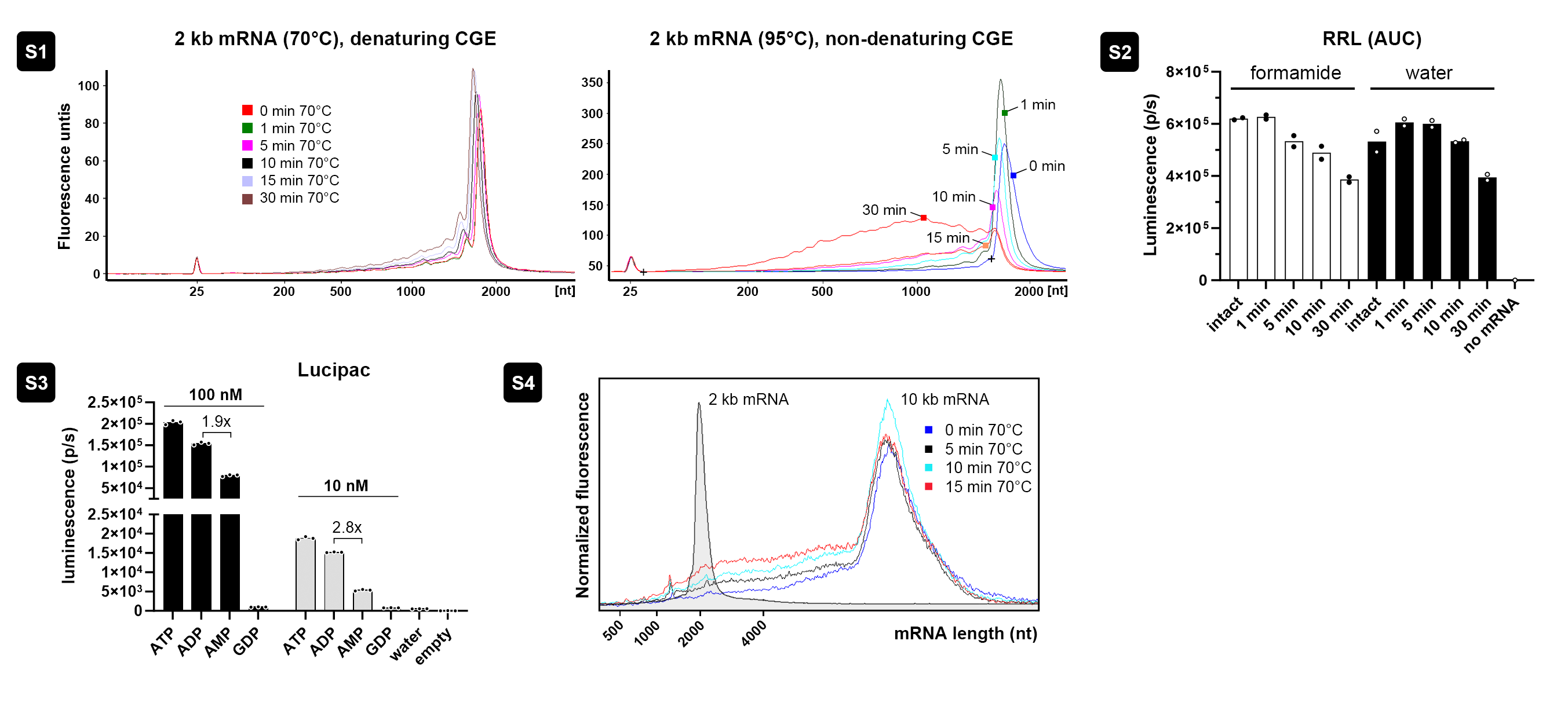

Supplement: Figure Supplement.tif [file KRNB_A_2363029_SM1796.tif]
